# Factors affecting food handling Practices among food handlers of Dangila town food and drink establishments, North West Ethiopia

**DOI:** 10.1186/1471-2458-14-571

**Published:** 2014-06-07

**Authors:** Ayehu Gashe Tessema, Kassahun Alemu Gelaye, Daniel Haile Chercos

**Affiliations:** 1 Department of Environmental and Occupational Health and Safety, Institute of Public Health, University of Gondar, Gondar, Ethiopia

**Keywords:** Knowledge of food hygiene, Personal hygiene, Food handling practices, Food handler, Working environment

## Abstract

**Background:**

Food borne diseases are major health problems in developed and developing countries including Ethiopia. The problem is more noticeable in developing countries due to prevailing poor food handling and sanitation practices, inadequate food safety laws, weak regulatory systems, lack of financial resources to invest on safer equipments, and lack of education for food handlers.

**Methods:**

The objective of this study was to assess food handling practice and associated factors among food handlers working in food and drinking establishments of Dangila town, North West Ethiopia. Cross-sectional quantitative study design was conducted among 406 food handlers working in 105 food and drink establishments from July to August 2013 in Dangila town. Data were collected using face to face interview with pretested structured questionnaire and physical observation.

**Result:**

The mean age of the respondents was 22.7 ± 4.2 years of which 62.8% of the food handlers were females. Two hundred thirteen (52.5%) of food handlers had good food handling practices. Marital status (AOR = 7.52, 95% CI, 1.45-38.97), monthly income (AOR = 0.395, 95% CI, 0.25-0.62), knowledge about food handling (AOR = 1.69, 95% CI, 1.05-2.73), existence of shower facility (AOR = 1.89, 95% CI, 1.12-3.21) and separate dressing room (AOR = 1.97, 95% CI, 1.11-3.49) were found to be significantly associated with good food handling Practices.

**Conclusion:**

Above half of food handlers had good food handling practices. Marital status, monthly income, knowledge status, existence of shower facility, existence of separate dressing room and presence of insect and rodent were factors associated with food handling Practices.

## Background

Food borne diseases are major health problems in developed and developing countries. The World Health Organization estimated that in developed countries, up to 30% of the populations suffer from food borne diseases each year, whereas in developing countries up to 2 million deaths are estimated per year [1]. Every day people all over the world get sick from the food they eat. This sickness is called food borne disease and is caused by dangerous microorganisms and/or toxic chemicals [2]. Millions of people become sick each year and thousands die after eating contaminated or mishandled foods [3]. Food handlers with poor personal hygiene working in food establishments could be potential sources of infections of many intestinal helminthes, protozoa, and pathogenic bacteria [4]. Food handler are anyone who works in a food and drink establishments and who handles food, or contact with any equipment or utensils that are likely to be in contact with food, such as cutlery, plates, bowls, or chopping boards [5].

In industrialized countries, infected food handlers are an important source of food borne disease. Ingestion of infected food can result in mild to severe illness, hospitalization or even death. Diseases with short incubation periods are more likely to be detected and attributed to infected food than those with longer incubation periods where the individual may not associate their illness with ingestion of infected food [6].

In Africa poverty is the underlying cause of consumption of unsafe food. Lack of access to potable water, poor government structural arrangement, communicable diseases, trade pressure, and inconvenient environmental conditions are notable reasons. High incidence of diarrheal diseases among children are an indications of the food hygiene situation in the African region [7].

There are many factors associated with food handling practices. A study done in Ankara, Turkey, Mekelle town, and Bahir Dar town, Ethiopia indicated that knowledge of food handling is significantly related with food handling practices [8-10], whereas, a study done on central India, Bangladesh, and Nigeria indicatedthat food handling practices was related with educational status of food handlers [11-13]. More ever, a study done in Nigeria and Kenya in 2009 showed that type of premise, unclean equipment and work responsibility was factors affecting food handling practices [1,14]. Gender was also found to be associated with food handling practices of vendors of street foods in Nairobi, Kenya [14]. In addition to socio demographic factors, environmental factors such as temperature, solid waste storage, solid waste disposal, latrine condition and hand washing facilities of the food and drink establishment were associated with food handling practices [1,9,15].

Food borne diseases are common in developing countries including Ethiopia because of the prevailing poor food handling and sanitation Practices, inadequate food safety laws, weak regulatory systems, lack of financial resources to invest safer equipments, and lack of education for food handlers. Taking in to consideration of Dangila town as a regional training center for various institutions in North Gojjam and in the town number of food and drink establishments are visible from time to time it is desirable to select town as a study area. In addition, there was no research done on this area which assesses the food handling practices of food handlers in the food and drink establishments of this particular town.

## Methods

### Study design

Cross-sectional quantitative study design was used to conduct this study.

### Study area and period

**Figure 1:**
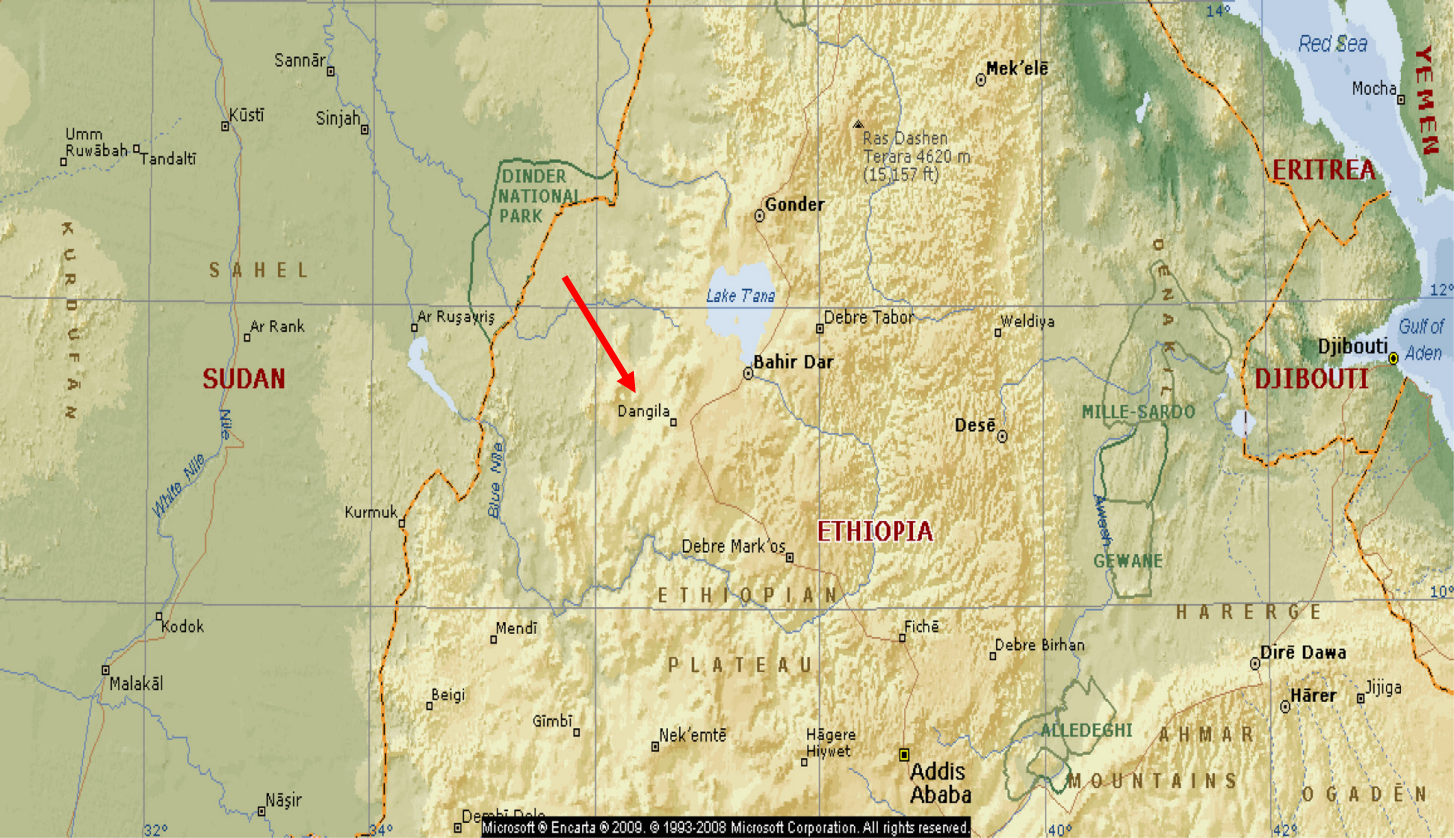
Map of Dangla town, Ethiopia; taken from microsoft encarta premium, 2009.

The study was conducted in Dangila town from July to August 2013. Dangila town is one of the largest and highly populated towns in Awi zone districts, Amhara regional state. It is about 80 km from regional capital, Bahir Dar and 485 km North West of the country capita Addis Ababa. Based on the annual report in 2012, the total population of Dangila town was 74,280. The town has 5 administrative kebeles. In the town, there are a total of 105 food and drink licensed establishments (29 hotels, 44 cafe and juice houses and 32 restaurants) (Figure 1) [16].

### Source population

All food handlers working in food and drink establishments, 430 in total, in the town were used as source of population.

### Inclusion and exclusion criteria

#### Inclusion criteria

All food and drink establishment in the town were included in the study.

Food handlers who were working in food and drink establishments during the study period were included.

#### Exclusion criteria

Food handlers who were unable to hear during data collection time were excluded from the study.

### Sample size

The sample size was calculated using a single population proportion formula. It was calculated taking 95% confidence interval, marginal error 5%, and good food handling Practices of food handlers as 63.9% [8].

The calculated sample size was 391. Since the final sample size calculated is more or less equal to the total food handlers in the town, which are 430, all food handlers are taken as the study subjects.

### Operational definitions

Practices**:** To assess the level of Practices, respondents were asked 17 questions from the questionnaire and those who scored ≤ the mean value were considered as having poor Practices and those who scored > the mean value were considered as having good Practices [10].

Knowledge: To assess the level of knowledge, respondents were asked 9 questions questionnaire and those who scored ≤ the mean value were considered as having poor knowledge and those who scored > the mean value were considered as having good knowledge [10].

### Data collection tools and procedures

A structured questionnaire and observational checklist was used for this study. For administering the interview and observation, six diploma holder nurses were recruited. Two supervisors who have BSc degree in Environmental health were also recruited for supervisory activities along with the principal investigator.

### Data quality control

The quality of data was assured by proper designing and pre-testing of the questionnaires. The questionnaire was prepared in English, translated to Amharic and then translated back to English to check for consistency. Training were given to data collectors and supervisors for two days on the objective, importance of the study, confidentiality of information, respondent’s right, techniques of interview, observation, and about pre-test.

Pretest was conducted on the neighboring district to ensure the validity and reliability of the survey tools and the necessary feedbacks were presented to data collectors.

The supervisors and the principal investigator monitored the data collection process to ensure the completeness and reliability of the gathered information throughout the data collection process.

### Data processing and analysis

The questionnaires was checked for completeness, cleaned and edited. Complete items were coded and entered onto Epi Info version 3.5.3 and transported to Statistical Package for the Social Science (SPSS) version 16 software packages for analysis. The results were presented in tables, figures and texts using descriptive statistics such as mean, standard deviation and percentage to describe the study population in relation to relevant variables. The data were analyzed using multiple logistic regressions to determine the effect of various factors on the outcome variable and to control confounding effect. Hosmer and Lemeshow goodness-of-fit test was found to be 0.414 and the method used was forward. The degree of association between independent and dependent variables were assessed using odds ratio with 95% confidence interval. Bivariate and multivariate logistic regression was applied, variables with P- value less than or equal to 0.2 at bivariate analysis were imported to multivariate analysis.

### Ethical consideration

Ethical approval and clearance was obtained from Institutional Review Board (IRB) of University of Gondar, Institute of Public Health. Permission was also obtained from the concerned bodies of Dangila municipality office and Dangila town administration health office. Written consent was obtained from owners/managers of the establishments but verbal for study subjects. Interview was carried out only with full consent of the person being interviewed. Each respondent was assured that the information provided by her/his was kept confidential and used only for the purpose of this research.

## Results

### Socio-demographic factors

A total of 406 food handlers working in 105 foods and drink establishments of Dangila town administration (responded to the questionnaire with 94.4% a response rate). The mean age of the respondent was 22.7 ± 4.2 years. Most of the food handlers, 374 (92.1%) were Orthodox by religion.

Two hundred fifty five (62.8%) of the respondents were females. More than two third 275 (67.5%) of the respondents were single, 279 (68.7%) were Amhara in their ethnicity. Half 202 (49.8%) of the respondents were attended primary school.

Regarding to the work responsibility of the study subjects; above half 234 (57.6%) were waiters. The majority of the respondents 362 (89.2%) had not taken food preparation and handling training. From trained respondents only 17 (4.2%) had certificate (Additional file 1).

### Food handling practices

Out of 406 food handlers working in food and drink establishments of Dangila town administration, 213 (52.5%) had good food handling Practices.

Among the total food handlers observed during visits, 319 (78.6%) used outer garments/gown of which 203 (63.6%) had a good food handling practices. Of the total food handlers who used outer garments, 283 (88.7%) of the food handlers outer garments were clean (Additional file 2).

### Environmental factors

Almost all food handlers, 400 (98.5%) and 397 (97.8%) were working in an establishment which had private pipe and toilet within the establishment respectively. Of the establishments which had toilet facility within the establishment, 262 (64.5%) were working in an establishment which had hand washing facility (Additional file 3).

### Knowledge level of food handlers

The majority of food handlers, 289 (71.2%) of the respondents, had poor knowledge score on food handling Practices. Most of food handlers 361(88.9%) had heard about food borne diseases of which 117 (32.4%) had a good knowledge. Among food handlers who believed that personal hygiene prevents food borne disease of which 117 (28.6%) had a good knowledge (Additional file 4).

### Association of different factors on food handling practices

In logistic regression analysis, each explanatory variable with outcome variable (food handling Practices) were assessed for its association. Variables with P-value above 0.2 were not exported to multivariate analysis.

The results of multivariate model revealed that marital status, monthly income, knowledge, presence of insects and rodents, existence of shower facility and separate dressing room were found to be significantly associated with food handling practices in multivariate analysis with P-value <0.05 (Additional file 5).

## Discussion

Marital status, monthly income, knowledge, presence of insects and rodents, existence of shower facility and separate dressing room were found to be factors affecting food handling practices in the study area.

In this study out of 406 food handlers working in food and drink establishments 213 (52.5%) had good food handling practices. This finding was consistent with studies in Malaysia and Nigeria, which had safety food handling practices of 54.7% and 54.7% [1,11] respectively. It was greater than findings in Turkey which had prevalence of 48.4% [9]. But this finding is lower than the finding in Mekelle town, Ethiopia, in which a practices of food handlers on food hygiene was found to be 63.9% [8]. The probable reasons for the differences might be due to difference in sociodemograhic and environmental factors difference in the two study groups.

Food handlers (respondents) those who were divorced 7.52 times more likely to had good food handling Practices (AOR = 7.52, 95% CI, 1.45-38.97) compared to those who were single. This finding suggests that the chance of getting good food handling practices among food handlers working in food and drink establishments is highest among food handlers whose marital status was divorced. The probable reason for this finding might be divorced food handlers might have experience of having good food handling practices during their marriage.

Food handlers whose monthly income < 379.00 ETB were 60.5% less likely to have good food handling practices compared to those whose monthly income ≥ 379.00 ETB (AOR = 0.395, 95% CI, 0.25-0.62). The possible reason for this might be those who had monthly income ≥379.00 ETB might have good educational status, experience and knowledge towards food handling practices.

Food handlers who had good knowledge were 1.69 times more likely to have good food handling practices compared to those who had poor knowledge (AOR = 1.69, 95% CI, 1.05-2.73).This finding is in line with the findings in Mekelle with [AOR: 3.61, 95% CI: (1.51-8.65] [8].

Food handlers who were working in an establishment which had shower facility were 1.89 times more likely to have good food handling practices compared to those who were working in an establishment which had no shower facility (AOR = 1.89, 95% CI, 1.12-3.21). The probable reason for this finding might be those food handlers working in food and drink establishments which had shower facility might better keep their personal hygiene and yield in good food handling practices.

Food handlers who were working in an establishment which had separate dressing room were 1.97 times more likely to have good food handling practices compared to those who were working in an establishment which had no separate dressing room (AOR = 1.97, 95% CI, 1.11-3.49). The possible reason for this finding might be those food handlers working in food and drink establishments which had separate dressing room may better keep clean their working environment and yield in good food handling practices.

Food handlers who were working in an establishment which had insects and rodents were 65% less likely to have good food handling practices compared to those who were working in an establishment which had no insects and rodents (AOR = 0.348, 95% CI, 0.196-0.617). The probable reason for this finding might be those food handlers working in food and drink establishments which had no insects and rodents may better keep their working environment from contamination and yield in good food handling practices.

## Conclusion

Above half of food handlers had good food handling practices. The predominant factors associated with good food handling practices were marital status, monthly income, knowledge status, existence of shower facility, existence of separate dressing room and presence of insects and rodents.

## Competing interests

The authors declare that they have no competing interests.

## Authors’ contributions

AGT was responsible for generating the concept of this research paper, literature review and organization, preparation of draft research proposal document, organizing data collection process, and preparation of draft data analysis and interpretation. KAG is participated in research topic preparation process, proposal research design process, data analysis, and interpretation process. DHC participated in proposal research design process, data analysis, and presentation and interpretation process of result, preparation of scientific paper or the manuscript, and corresponding author of the manuscript. All authors read and approved the final manuscript.

## Pre-publication history

The pre-publication history for this paper can be accessed here:

http://www.biomedcentral.com/1471-2458/14/571/prepub

## Supplementary Material

Additional file 1: Table S1 Socio-demographic characteristics of food handlers working in food and drink establishments of Dangila town administration, Amhara region, North West Ethiopia, 2013. Click here for file

Additional file 2: Table S2 Results of observational checklists to assess food handling Practices among food handlers working in food and drink establishments in Dangila town adminestration, Awi, Northwest Ethiopia, 2013. Click here for file

Additional file 3: Table S3 Environmental characteristics of food handling Practices among food handlers working in food and drink establishments of Dangila town adminestration, Amhara region, Northwest Ethiopia, 2013. Click here for file

Additional file 4: Table S4 Knowledge status of food handlers on food handling Practices working in food and drink establishements in Dangila town adminestration, Amhara region, Northwest Ethiopia, 2013. Click here for file

Additional file 5: Table S5 Multivariate logistic regression results on factors associated with food handling Practices among food handlers working in food and drink establishments in Dangila town, Awi zone, Northwest Ethiopia, 2013. Click here for file
