# Supplementary material for: Factors affecting food handling Practices among food handlers of Dangila town food and drink establishments, North West Ethiopia
Source: BMC Public Health. 2014 Jun 7;14:571. doi: 10.1186/1471-2458-14-571 (PMC4057591; doi:10.1186/1471-2458-14-571)
Supplement: Additional file 1: Table S1 — Socio-demographic characteristics of food handlers working in food and drink establishments of Dangila town administration, Amhara region, North West Ethiopia, 2013. [file 1471-2458-14-571-S1.doc]

**Additional file 1: Table S1** Socio-demographic characteristics of food handlers working in food and drink establishments of Dangila town administration, Amhara region, North West Ethiopia, 2013

| Variables | Number (n = 406) | Percent (%) |
| --- | --- | --- |
| Sex | | |
| Male | 151 | 37.2 |
| Female | 255 | 62.8 |
| Age | | |
| ≤20 years | 139 | 34.2 |
| 21-30 years | 250 | 61.6 |
| 31-40 years | 14 | 3.4 |
| >40 years | 3 | 0.7 |
| Educational status of food handler | | |
| Unable to read and write | 25 | 6.2 |
| Primary school | 202 | 49.8 |
| Secondary school | 137 | 33.7 |
| College and above | 42 | 10.3 |
| Marital status of food handler | | |
| Single | 275 | 67.7 |
| Married | 118 | 29.1 |
| Divorced | 13 | 3.2 |
| Religion of food handler | | |
| Orthodox | 374 | 92.1 |
| Muslim | 21 | 5.2 |
| Protestant | 11 | 2.7 |
| Ethnicity of food handler | | |
| Awi | 120 | 29.6 |
| Amhara | 279 | 68.7 |
| Others | 7 | 1.7 |
| Monthly income of food handler | | |
| ≥ 379 ETB | 242 | 59.6 |
| < 379 ETB | 164 | 40.4 |
| Work responsibility of food handler | | |
| Cook | 152 | 37.4 |
| Waiter | 234 | 57.6 |
| Washer | 20 | 4.9 |
| How long you stay in this work | | |
| < 2 years | 230 | 56.7 |
| ≥ 2 years | 176 | 43.3 |
| Did you have training on food preparation and handling | | |
| Yes | 44 | 10.8 |
| No | 362 | 89.2 |
| If “yes” Do you have certificate (n = 44) | | |
| Yes | 17 | 4.2 |
| No | 27 | 6.7 |
