# Supplementary material for: Factors affecting food handling Practices among food handlers of Dangila town food and drink establishments, North West Ethiopia
Source: BMC Public Health. 2014 Jun 7;14:571. doi: 10.1186/1471-2458-14-571 (PMC4057591; doi:10.1186/1471-2458-14-571)
Supplement: Additional file 2: Table S2 — Results of observational checklists to assess food handling Practices among food handlers working in food and drink establishments in Dangila town adminestration, Awi, Northwest Ethiopia, 2013. [file 1471-2458-14-571-S2.doc]

**Additional file 2: Table 2** Results of observational checklists to assess food handling Practices among food handlers working in food and drink establishments in Dangila town adminestration, Awi , Northwest Ethiopia, 2013

| Variables | Total | Practices Status | |
| --- | --- | --- | --- |
|  |  | Good | Poor |
| Wearing outer garments/gown during visit | | | |
| Yes | 319(78.6%) | 203(63.6%) | 116(36.4%) |
| No | 87(21.4%) | 10(11.5%) | 77(88.5%) |
| If “yes” Cleanness of outer garments/gown (n = 319) | | | |
| Yes | 283(88.7%) | 199(70.3%) | 84(29.7%) |
| No | 36(11.3%) | 4(11.1%) | 34(88.9%) |
| Hair covered while working | | | |
| Yes | 207(51%) | 147(71%) | 60(29%) |
| No | 199(49%) | 66(33%) | 133(67%) |
| Finger nail short trimmed and clean | | | |
| Yes | 363(89.4%) | 206(56.7%) | 157(43.3) |
| No | 43(10.6%) | 7(16.3%) | 36(84.7) |
| Wear any jewelry or ring on hand at time of visit | | | |
| Yes | 160(39.4%) | 77(48.1%) | 83(51.9%) |
| No | 246(60.6%) | 136(55.3%) | 110(44.7%) |
| Clean the work surfaces after each task | | | |
| Yes | 400(98.5%) | 212(53%) | 188(47%) |
| No | 6(1.5%) | 1(16.7%) | 5(83.3%) |
| Used soap/ detergent for washing dishes | | | |
| Yes | 403(99.3%) | 212(52.6%) | 191(48.4%) |
| No | 3(0.7%) | 1(33.3%) | 2(66.7%) |
| Used hot water for washing dishes | | | |
| Yes | 214(52.7%) | 169(79%) | 45(21%) |
| No | 192(47.3%) | 44(22.9%) | 148(77.1) |
| Wash their utensils using three washing compartments | | | |
| Yes | 320(78.8%) | 208(65%) | 112(35%) |
| No | 86(21.2%) | 5(5.8%) | 81(19%) |
| Wash chopping board/knife with soap/belch after using | | | |
| Yes | 375(92.4%) | 208(55.5%) | 167(44.4%) |
| No | 31(7.6%) | 5(16%) | 26(84%) |
| Wash his/her hands with soap and water before working with food | | | |
| Yes | 390(96.1%) | 213(54.6) | 177(45.4%) |
| No | 16(3.9%) | 0(0%) | 16(100%) |
| Wash his/her hands with soap and water after visiting a latrine | | | |
| Yes | 387(95.3%) | 212(54.8) | 175(45.2%) |
| No | 19(4.7%) | 1(5.3%) | 18(94.7%) |
| Kept ready-to-eat foods in a clean container and covered properly | | | |
| Yes | 390(96.1%) | 209(53.6%) | 181(46.4%) |
| No | 16(3.9%) | 4(25%) | 12(75%) |
| Stored food utensils in well-arranged manner in shelf or cupboard | | | |
| Yes | 389(95.8%) | 211(54.2%) | 178(45.8%) |
| No | 17(4.2%) | 2(11.8%) | 15(88.2%) |
| Store raw food item in an area separate from cooked food | | | |
| Yes | 394(97%) | 210(53.3%) | 184(46.7%) |
| No | 12(3%) | 3(25%) | 9(75%) |
| Stored perishable ready- to- eat foods in refrigerator | | | |
| Yes | 247(60.8%) | 172(69.6%) | 75(25%) |
| No | 159(39.2%) | 41(25.8%) | 118(74.2%) |
| Took medical checkup in the past six months | | | |
| Yes | 97(23.9%) | 80(82.5) | 17(17.5%) |
| No | 309(76.1%) | 133(43%) | 176(57%) |
