# Supplementary material for: Factors affecting food handling Practices among food handlers of Dangila town food and drink establishments, North West Ethiopia
Source: BMC Public Health. 2014 Jun 7;14:571. doi: 10.1186/1471-2458-14-571 (PMC4057591; doi:10.1186/1471-2458-14-571)
Supplement: Additional file 3: Table S3 — Environmental characteristics of food handling Practices among food handlers working in food and drink establishments of Dangila town adminestration, Amhara region, Northwest Ethiopia, 2013. [file 1471-2458-14-571-S3.doc]

**Additional file 3: Table S3** Environmental characteristics of food handling Practices among food handlers working in food and drink establishments of Dangila town adminestration, Amhara region , Northwest Ethiopia, 2013

| Variables | Number (n = 406) | Percent (%) |
| --- | --- | --- |
| Source of water | | |
| Pipe from neighbors | 6 | 1.5 |
| Private pipe | 400 | 98.5 |
| Availability of toilet facility | | |
| No | 9 | 2.2 |
| Yes | 397 | 97.8 |
| If “yes” hand washing facility (n = 397) | | |
| No | 135 | 33.3 |
| Yes | 262 | 64.5 |
| Availability of container for solid waste | | |
| No | 10 | 2.5 |
| Yes | 396 | 97.5 |
| Type of solid waste storage (n = 396) | | |
| Dust bin | 155 | 38.2 |
| Barrel | 48 | 11.8 |
| Suck | 173 | 42.6 |
| Other | 20 | 4.9 |
| Means for waste water from hand washing basin, and dishwashing facilities disposed off | | |
| Open field | 14 | 3.4 |
| Septic tank | 50 | 12.3 |
| Latrine | 2 | 0.5 |
| Storm water drainage | 63 | 15.5 |
| Seepage pit | 275 | 67.7 |
| Other | 2 | 0.5 |
| Existence of insects and rodents in the establishment | | |
| No | 327 | 80.5 |
| Yes | 79 | 19.5 |
| Existence of kitchen in the establishment | | |
| No | 9 | 2.2 |
| Yes | 397 | 97.8 |
| The kitchen has wall and ceiling free from dust, spider webs and smoke particles (n = 397) | | |
| No | 44 | 10.8 |
| Yes | 353 | 86.9 |
| Availability of shower facility | | |
| No | 312 | 76.8 |
| Yes | 94 | 23.2 |
| Separate room for dressing of food handlers | | |
| No | 329 | 81 |
| Yes | 77 | 19 |
| Inspection of the establishment | | |
| No | 44 | 10.8 |
| Yes | 362 | 89.2 |
