# Supplementary material for: Factors affecting food handling Practices among food handlers of Dangila town food and drink establishments, North West Ethiopia
Source: BMC Public Health. 2014 Jun 7;14:571. doi: 10.1186/1471-2458-14-571 (PMC4057591; doi:10.1186/1471-2458-14-571)
Supplement: Additional file 4: Table S4 — Knowledge status of food handlers on food handling Practices working in food and drink establishements in Dangila town adminestration, Amhara region, Northwest Ethiopia, 2013. [file 1471-2458-14-571-S4.doc]

**Additional file 4: Table S4** Knowledge status of food handlers on food handling Practices working in food and drink establishements in Dangila town adminestration, Amhara region , Northwest Ethiopia, 2013

| Variables | Total | Knowledge status | |
| --- | --- | --- | --- |
|  |  | Good | Poor |
| Heard about food borne disease | | | |
| No | 45(11.1%) | 0(0%) | 45(100%) |
| Yes | 361(88.9%) | 117(32.4%) | 244(67.6%) |
| Cause of food borne disease | | | |
| Contaminated with bacteria & parasites | 224(55.2%) | 113(50.5%) | 111(49.5%) |
| Adding chemicals | 85(20.2%) | 43(50.6%) | 42(49.4%) |
| Anger of God | 3(0.7%) | 1(33.3%) | 2(66.7%) |
| Unhygienic food preparation | 196(48.3%) | 89(45.4%) | 107(54.6%) |
| Improper food handling Practices | 58(14.3%) | 43(74.1%) | 15(25.9%) |
| un approved sources | 98(24.3%) | 40(40.8%) | 58(59.2%) |
| Mode of transmission of food borne disease | | | |
| Contaminated food | 238(58.6%) | 113(47.5%) | 125(52.5%) |
| Contaminated water | 201(49.5%) | 97(48.3%) | 104(51.7%) |
| Infected food handlers | 146(36%) | 69(47.3%) | 77(52.7%) |
| Vectors | 47(11.6%) | 34(72.3%) | 13(26.7%) |
| Reason for food contamination | | | |
| Contact of unhygienic hands | 259(58.6%) | 108(41.7%) | 151(58.3%) |
| Unhygienic working environment | 205(50.5%) | 64(31.2%) | 141(68.8%) |
| Using of contaminated water | 159(39.2%) | 63((39.6%) | 96(40.4%) |
| Unclean utensils | 188(46.3%) | 90(47.9%) | 98(52.1%) |
| Infected food handlers | 50(12.6%) | 20(40%) | 30(60%) |
| Exposure to insects and rats | 67(16.5%) | 13(19.4%) | 54(80.6%) |
| Danger temperature zone for potentially hazardous foods | | | |
| Below 5 c^o^ | 22(5.4%) | 3(13.6%) | 19(86.4%) |
| 5-60 c^o^ | 37(9.1) | 26(70.3%) | 11(29.7%) |
| Above 60 c^o^ | 6(1.5%) | 0(0%) | 6(100%) |
| I do not know | 341(84%) | 88(25.8%) | 253(74.2%) |
| Raw milk transmits disease | | | |
| Yes | 332(81.8%) | 113(34%) | 219(66%) |
| No | 59(14.5%) | 0(0%) | 59(100%) |
| I do not know | 15(3.7%) | 4(26.7%) | 11(73.3%) |
| Raw meat transmit disease | | | |
| Yes | 379(93.3%) | 116(30.6) | 263(69.4%) |
| No | 24(5.9%) | 1(4.2%) | 23(95.8%) |
| I do not know | 3(0.7%) | 0(0%) | 3(100%) |
| Do raw vegetables transmit disease | | | |
| Yes | 266(65.5%) | 104(39%) | 162(61%) |
| No | 120(29.6%) | 11(9.2%) | 109(90.8%) |
| I do not know | 20(4.9%) | 2(10%) | 18(90%) |
| Good personal hygiene prevents food borne disease | | | |
| Yes | 377(92.9%) | 117(28.6%) | 260(64.4%) |
| No | 19(4.7%) | 0(0%) | 19(100%) |
| I do not know | 10(2.5%) | 0(0%) | 10(100%) |
