# Supplementary material for: Factors affecting food handling Practices among food handlers of Dangila town food and drink establishments, North West Ethiopia
Source: BMC Public Health. 2014 Jun 7;14:571. doi: 10.1186/1471-2458-14-571 (PMC4057591; doi:10.1186/1471-2458-14-571)
Supplement: Additional file 5: Table S5 — Multivariate logistic regression results on factors associated with food handling Practices among food handlers working in food and drink establishments in Dangila town, Awi zone, Northwest Ethiopia, 2013. [file 1471-2458-14-571-S5.doc]

**Additional file 5: Table S5** Multivariate logistic regression results on factors associated with food handling Practices among food handlers working in food and drink establishments in Dangila town, Awi zone, Northwest Ethiopia, 2013

| Variable | FHP | | COR (95% CI) | AOR (95% CI) |
| --- | --- | --- | --- | --- |
|  | Good | Poor |  |  |
| Marital status | | | | |
| Single | 137 | 138 | 1.00 | 1.00 |
| Married | 74 | 44 | 0.590 (0.380-0.918) |  |
| Divorced | 2 | 11 | 5.46 (1.18-25.09) | 7.52 (1.45-38.97)** |
| Monthly income | | | | |
| ≥379 ETB | 103 | 139 | 1.00 | 1.00 |
| <379 ETB | 110 | 54 | 0.364 (0.24-0.55) | 0.395 (0.25-0.62)* |
| Knowledge status | | | | |
| Poor | 165 | 124 | 1.00 | 1.00 |
| Good | 48 | 69 | 1.91 (1.23-2.95) | 1.69 (1.05-2.73)*** |
| Existence of shower facility | | | | |
| No | 179 | 133 | 1.00 | 1.00 |
| Yes | 34 | 60 | 2.37 (1.47-3.82) | 1.89 (1.12-3.21)** |
| Existence of separate dressing room | | | | |
| No | 184 | 145 | 1.00 | 1.00 |
| Yes | 29 | 48 | 2.1 (1.26-3.49) | 1.97 (1.11-3.49)*** |
| Presence of insects and rodents | | | | |
| No | 159 | 168 | 1.00 | 1.00 |
| Yes | 54 | 25 | 0.438 (0.26-0.738) | 0.348 (0.196-0.617)* |

Key

* = variables with p-value <0.001.

** = variables with p-value <0.02.

*** = variables with p-value <0.04.
